# The sensitivity of photosynthesis to magnesium deficiency differs between rice (*Oryza sativa* L.) and cucumber (*Cucumis sativus* L.)

**DOI:** 10.3389/fpls.2023.1164866

**Published:** 2023-04-14

**Authors:** Xusheng Meng, Song Bai, Shiyu Wang, Yonghui Pan, Kehao Chen, Kailiu Xie, Min Wang, Shiwei Guo

**Affiliations:** ^1^ Jiangsu Provincial Key Lab of Solid Organic Waste Utilization, Jiangsu Collaborative Innovation Center of Solid Organic Wastes, The Key Laboratory of Plant Immunity, Nanjing Agricultural University, Nanjing, China; ^2^ Rice Research Institute, Guangdong Academy of Agricultural Sciences, Guangzhou, China; ^3^ School of Land Resources and Environment, Jiangxi Agricultural University, Nanchang, China

**Keywords:** magnesium, rice, cucumber, photosynthesis, photoprotection, NPQ

## Abstract

Magnesium is an essential macronutrient for plant photosynthesis, and in response to Mg deficiency, dicots appear more sensitive than monocots. Under Mg deficiency, we investigated the causes of differing photosynthetic sensitivities in a dicot and a monocot species. Rice (*Oryza sativa* L.) and cucumber (*Cucumis sativus* L.) were grown in hydroponic culture to explore their physiological responses to Mg deficiency stress. Both Mg-deficient rice and cucumber plants exhibited lower biomass, leaf area, Mg concentration, and chlorophyll content (Chl) compared with Mg-sufficient plants. However, a more marked decline in Chl and carotenoid content (Car) occurred in cucumber. A lower CO_2_ concentration in chloroplasts (*C*_c_) was accompanied by a decrease in the maximum rate of electron transport (*J*_max_) and the maximum rate of ribulose 1,5-bisphosphate carboxylation (*V*_cmax_), restricting CO_2_ utilization in Mg-deficient plants. Rice and cucumber photorespiration rate (*P*_r_) increased under Mg deficiency. Additionally, for cucumber, Car and non-photochemical quenching (NPQ) were reduced under lower Mg supply. Meanwhile, cucumber Mg deficiency significantly increased the fraction of absorbed light energy dissipated by an additional quenching mechanism (Φf,D). Under Mg deficiency, suppressed photosynthesis was attributed to comprehensive restrictions of mesophyll conductance (*g*_m_), *J*_max_, and *V*_cmax_. Cucumber was more sensitive to Mg deficiency than rice due to lower NPQ, higher rates of electron transport to alternative pathways, and subsequently, photooxidation damage.

## Introduction

Magnesium (Mg) is vital for plant growth and reproductive success as it is an irreplaceable component of cells and tissues (Zhao et al., 2012), with a total Mg cellular concentration of 15-25 mM with 15%–20% bound to chlorophyll and free magnesium concentration frequently at less than the millimolar level. Mg mainly participates in photosynthesis, energy metabolism, and nucleic acid and protein synthesis. Moreover, some Mg is allocated to the cell walls by combining with pectin (Cowan, 2002; Shaul, 2002). Because of cation antagonism, Mg^2+^ plant uptake can be strongly depressed by NH_4_^+^ and K^+^. Furthermore, an unbalanced K/Mg ratio inhibits photosynthesis and N metabolism (Xie et al., 2021). Therefore, excessive chemical fertilizer applications with low Mg supply increase Mg deficiency (Wilkinson et al., 1990; Gransee and Fuhrs, 2013). Plants require enough Mg to allow biomass formation and carbohydrate partitioning; many studies have shown lower dry matter of various plants under Mg deficiency (Ding et al., 2006; Ceylan et al., 2016; Huang et al., 2016; Trankner et al., 2016). The root-to-shoot ratio can also be downregulated during the early stage of Mg deficiency (Fischer and Bremer, 1993), as Mg is necessary for sucrose phloem loading. Mg deficiency suppresses crop yields by reducing plant photosynthetic rate and carbohydrate transportation, ultimately restricting agricultural production (Farhat et al., 2016).

Mg in plants is preferentially allocated in the chloroplasts for photosynthetic processes (Chen et al., 2018). Mg deficiency reduces the photosynthetic rate through light reaction and CO_2_ assimilation since Mg is not only a chlorophyll structural component but also the activator of numerous photosynthetic enzymes (Schneider et al., 1992). Chlorophyll acts in pigment–protein complexes to harvest photons in photosystems II and I (PSII and PSI) (Jansson, 1994), but Mg deficiency restrains chlorophyll synthesis and promotes chlorophyll degradation (Hermans et al., 2010), subsequently reducing the photosynthetic electron transport rate (*J*) (Laing et al., 2000). Ribulose 1,5-bisphosphate carboxylase (Rubisco), a dominant enzyme in CO_2_ assimilation, is regulated by Mg concentration in chloroplast stroma (Lorimer et al., 1976). Additionally, reduced CO_2_ diffusion leads to downregulated chloroplast CO_2_ concentration (Fischer and Bremer, 1993; Hariadi and Shabala, 2004), which affects the carboxylation reaction rate. Mg is also involved in photoprotection. For example, the maximum quantum efficiency of PSII (*F*_v_/*F*_m_) declined in various plant species under Mg-deficient conditions (Laing et al., 2000; Hariadi and Shabala, 2004; Yang et al., 2012). Reduced *F*_v_/*F*_m_ means photoinhibition occurs under Mg deficiency, which is induced by failed D1 protein repair in the PSII reaction center (Traenkner et al., 2018).

Interveinal chlorosis in the leaves may be attributed to Mg-deficient-induced photooxidation damage. Once photosynthesis is suppressed, the flow of electrons transferred to O_2_ increases to generate reactive oxygen species (ROS) such as superoxide radicals (O_2_^·−^), hydrogen peroxide (H_2_O_2_), and hydroxyl radicals (OH^·^) (Cakmak and Kirkby, 2008). Abiotically stressed plants inevitably absorb excessive light energy and, therefore, have evolved several physiological processes to minimize injury, such as strengthening the photorespiration rate (*P*_r_) and non-photochemical quenching (NPQ) (Demmig-Adams and Adams, 2002); elevating the electron transfer rates to some additional/alternative electron transport pathways (Asada, 1999; Cournac et al., 2000); enhancing the antioxidative defense system by increasing the activities of enzymes including SOD, CAT, and POD (Ding et al., 2008); and promoting the D1 protein turnover (a core component of the PSII reaction center) resynthesis (Zhang et al., 2000). However, the defense system is destroyed under magnesium deficiency, and enhanced ROS contributes to lipid peroxidation and chlorophyll degradation (Wingler et al., 2005).

Structures and intracellular characters vary between monocots and dicots. For example, a particular cell wall structure is found in commelinid monocots which possess less pectin in cell walls than non-commelinid monocots and dicots (White et al., 2018). Different pectin content between plant groups might affect Mg allocation at the cellular level, as some Mg is bound to pectin (Marschner, 1995). Dicots appear more sensitive than monocots in response to Mg deficiency. Meta-analysis has demonstrated that critical Mg concentrations for net photosynthetic rate (*P*_n_) in monocots are lower than in dicots (Hauer-Jakli and Trankner, 2019). Wang et al. (2020b) found that different agronomic efficiencies of Mg fertilizers across crop species were due to variations in Mg uptake or utilization; vegetables (dicots) were always the most responsive to Mg applications, and cereals (monocots) the least. However, although these findings have proven sensitivity differences between dicots and monocots, the inherent cause is still unknown.

In the present study, rice and cucumber were grown in hydroponic culture to compare their physiological responses to magnesium deficiency. In a preliminary experiment, we found that cucumber leaves developed necrosis, while rice leaves stayed green while being supplied with low Mg concentration (0.01 mM, data not shown). Our primary objectives were to 1) compare the physiological and photosynthetic differences between rice and cucumber under Mg deficiency, 2) reveal the underlying mechanisms of photosynthesis downregulation under Mg deficiency, and (3) investigate the cause(s) of differing sensitiveness between the two plant species. Our results will provide insight into the mechanisms underlying the effects of Mg deficiency on plant growth and photosynthesis and increase our knowledge of specific Mg nutrient management on different crops, which is vital to maintain sustainable agricultural development.

## Materials and methods

### Plant materials and culture conditions

Rice (*Oryza sativa* L. cv. Shanyou 63) and cucumber (*Cucumis sativus* L. cv. Jinchun 4) were grown in a greenhouse. The greenhouse has a stable environment, provided with a 14-h photoperiod, a constant relative humidity of 40%∼60%, a photosynthetic photon flux density (PPFD) of 400 μmol m^−2^ s^−1^, and a day/night temperature of 30°C/25°C. Rice seeds were sterilized in 10% H_2_O_2_ for 1 h and then germinated in moist gauze. Cucumber seeds were soaked in water for 1 h and germinated in sterile quartz sand. After a preculture for 2 weeks, the uniform seedlings with 3 visible leaves were transferred to 6.5 L containers with a quarter-strength nutrient solution for the first 4 days, then transferred to a half-strength nutrient solution for another 4 days before providing a full-strength nutrient solution. Rice and cucumber seedlings were then supplied with two Mg-deficient treatments (Mg0.01, 0.01 mmol L^−1^; Mg0.1, 0.1 mmol L^−1^) and one Mg-sufficient treatment (Mg1, 1 mmol L^−1^), and Mg was provided by MgSO_4_·7H_2_O. The nutrient composition of the full-strength culture solutions was as follows: 2.86 mM of N provided by equimolar amounts of (NH_4_)_2_SO_4_ and Ca(NO_3_)_2_, 1.43 mM of Ca provided by equimolar amounts of Ca(NO_3_)_2_ and CaCl_2_, 0.32 mM of P and 1.03 mM of K provided by KH_2_PO_4_ and K_2_SO_4_, 35.8 μM of Fe-EDTA, 9.10 μM of MnCl_2_·4H_2_O, 0.52 of μM (NH_4_)_6_Mo_7_O_24_·4H_2_O, 18.5 μM of H_3_BO_3_, 0.15 μM of ZnSO_4_·7H_2_O, 0.16 μM of CuSO_4_·5H_2_O, and 0.1 μM of Na_2_SiO_3_·9H_2_O. Dicyandiamide was added to each nutrient solution to prevent ammonium oxidation. The nutrient solutions were renewed every 4 days, and the pH was adjusted to 5.5 ± 0.1 every day with HCl (1 M) and NaOH (1 M).

### Gas exchange and photochemical measurements

Gas exchange and photochemical measurements were determined on the second fully expanded leaves by a portable photosynthesis system (LI-6400, LI-COR Inc., Lincoln, NE, USA) equipped with an integrated fluorescence leaf chamber. Four leaves of each treatment were selected to measure from 9:00 to 14:00, set with a PPFD of 1,500 µmol m^−2^ s^−1^, a CO_2_ concentration of 400 μmol mol^−1^, a relative humidity of 40%~60%, and a leaf temperature of 30°C. The gas exchange parameters, steady-state fluorescence (*F*_s_), and maximum fluorescence (*F*′_m_) were recorded when stability was achieved after equilibration (approximately 20 min after clamping the leaf). The minimal level of fluorescence (*F*_o_) and the maximal fluorescence level (*F*_m_) of the leaves were measured from 2:00 to 3:00 after dark adaption sufficiently.

The actual photochemical efficiency of photosystem II (ΦPSII), the proportion of thermally dissipated energy through NPQ (ΦNPQ), the fraction of absorbed light energy dissipated by additional quenching mechanism (Φf,D) (Hendrickson et al., 2004), the electron transport rate (*J*), and the excess of photosynthetic linear electron transport not used for carbon assimilation (*J*_excess_) were calculated as follows (Streb et al., 2005; Savitch et al., 2010):


$$\text{ΦPSII}=\frac{F'\text{m}-F\text{s}}{F'm}$$

$$\text{ΦNPQ}=\frac{Fs}{F'\text{m}}\;-\frac{Fs}{Fm}$$

$$\text{Φf,D}=\frac{F\text{s}}{F\text{m}}$$

$$J=\text{ΦPSII}\times \text{ PPFD}\times \alpha_{leaf}\times \beta$$

$$J_{excess}=J-4\times \left(P_{n}+R_{d}\right)$$

Where *α*_leaf_ is the leaf absorbance and *β* reflects the partitioning of the absorbed quanta between PSII and PSI, and *R*_d_ is the day respiration rate. Light response curves and ΦPSII were measured in gas (98% N_2_ and 2% O_2_) at nine levels of PPFD (2,000, 1,500, 1,000, 500, 200, 150, 100, 50, 0 μmol m^−2^ s^−1^) by the portable photosynthesis system. The slope of the relationship between ΦPSII and 4ΦCO_2_ (the quantum efficiency of CO_2_ uptake) is calculated to be the value of α_leaf_ × β (Valentini et al., 1995).

The variable *J* method proposed by Harley et al. (1992). was used to calculate mesophyll conductance (*g*_m_) and CO_2_ concentration in chloroplasts (*C*_c_) as follows:


$$g_{m}=\frac{P_{n}}{\frac{C_{i}-\Gamma^{*}(J+8(P_{n}+R_{d}))}{J-4(P_{n}+R_{d})}}$$

$$C_{c}=C_{i}-\frac{P_{n}}{g_{m}}$$

The CO_2_ compensation point in the absence of mitochondrial respiration (Γ*) and *R*_d_ were measured through the Laisk method, as reported by Brooks and Farquhar (1985).

The maximum quantum efficiency of photosystem II (*F*_v_/*F*_m_) and the NPQ were computed as follows:


$$F_{v}/F_{m}=\frac{F_{m}-F_{o}}{F_{m}}$$

$$\text{NPQ}=\frac{F_{m}-F{'}_{m}}{F{'}_{m}}$$

After the above measurements of photosynthetic parameters, three rice and three cucumber leaves were selected to conduct light and CO_2_ response curves. Relative humidity and leaf temperature inside the measurement chamber were kept as described before. Light response curves were conducted by adjusting PPFD to nine levels (2,000, 1,500, 1,000, 500, 200, 150, 100, 50, 0 μmol m^−2^ s^−1^) at a constant CO_2_ partial pressure (400 μmol mol^−1^). For CO_2_ response curves, measurements were determined by adjusting CO_2_ concentrations to 11 levels (400, 300, 200, 150, 100, 50, 400, 600, 800, 1,000, and 1,200 μmol mol^−1^) at a constant PPFD (1,500 μmol m^−2^ s^−1^). All the gas exchange parameters of the leaves were recorded when stability was achieved at the corresponding ambient environments. The slope of the linear part of the light response curve (0, 50, 100, 150, 200 μmol m^−2^ s^−1^) and the CO_2_ response curve (50, 100, 150, 200 μmol mol^−1^) was calculated as apparent quantum yield (*α*) and carboxylation efficiency (CE). The maximum rate of RuBP carboxylation (*V*_cmax_) and the maximum rate of RuBP regeneration (*J*_max_) were evaluated by the CO_2_ response curve fitting model provided by Sharkey et al. (2007).

CO_2_ concentration in the chloroplasts at which the transition from Rubisco to RuBP regeneration limitation occurs (*C*_trans_) was calculated as follows:


$$C_{trans}=\frac{K_{c}(1+0/K_{o})J/4V_{cmax}-2\Gamma^{*}}{1-J/4V_{c\max}}$$

Rubisco kinetics parameters were according to Sharkey et al. (2007): *K*_c_ = 27.24 Pa, *K*_o_ = 16.58 kPa, and *O* = 21 kPa.

### Photorespiration rate measurement

Photorespiration rate measurements were also determined on the second fully expanded leaves by a portable photosynthesis system, which was connected to a gas containing 98% N_2_ and 2% O_2_; FFPD, CO_2_ concentration, relative humidity, and leaf temperature were maintained as described in the measurement of *P*_n_, and the net photosynthetic rate in 2%O_2_ (*P*_n_2%O_2_) was recorded when stability was achieved after equilibration.

The photorespiration rate (*P*_r_) was computed as follows:


$$P_{r}=P_{n}2\%\;O_{2}-P_{n}$$

### Dynamic measurements of photosynthetic parameters

Considering that the appearance of Mg-deficient symptoms in the plant leaves was a slow process and the leaves of cucumber became necrotic gradually, a transient measurement of photosynthesis could not exhibit the conditions of the leaves in this process; hence, the uppermost expanded leaves were selected to conduct a 7-day dynamic measurement. *P*_n_, *F*_v_/*F*_m_, and NPQ were measured every 2 days as described before.

### Determination of chlorophyll, carotenoid, and Mg contents and biomass

After the measurements of photosynthetic parameters, 0.2 g of fresh second fully expanded leaves were cut into little pieces and extracted with 20 ml of alcohol (95%, v/v). After a 24-h extraction in a dark place, the absorbance of the extract was measured by a spectrophotometer at 665, 649, and 470 nm, and then the chlorophyll and carotenoid contents were calculated from those three absorbances. Another part of these leaves was used to determine leaf mass per area (LMA) and Mg contents. Leaves were shot for leaf area estimation, and LMA was calculated by dividing leaf dry matter by leaf area concentrated. Fresh leaves were dried under 80°C to a constant weight, then weighed and digested in mixed HNO_3_ and HClO_4_. The concentrations of Mg were determined by ICP-OES (Agilent 710, Agilent Technologies, USA). Four plants in each treatment were also dried under 80°C to constant weight to determine biomass.

### Determination of the activities of ROS-scavenging enzymes

Fresh second fully expanded leaf (0.2 g) was precooled by liquid nitrogen and ground with 2 ml of cold phosphate buffer (50 mM, pH 7.8) containing insoluble polyvinylpyrrolidone (1%) and ethylenediaminetetraacetic acid (0.2 mM). The homogenate was centrifuged at 10,000*g* for 15 min at 4°C, and then the supernatant was collected for enzymatic activity analysis. SOD, POD, and CAT activities were determined by the methods described by Wang et al. (2020a).

### Statistical analysis

ANOVA with Duncan’s multiple range test was conducted on all measured parameters to identify the difference between Mg treatments using SPSS 18.0 software. Graphics and regression analysis were conducted using OriginPro 8.5 software and Adobe Illustrator 2020.

## Results

### Rice and cucumber physiological traits

**Figure 1:**
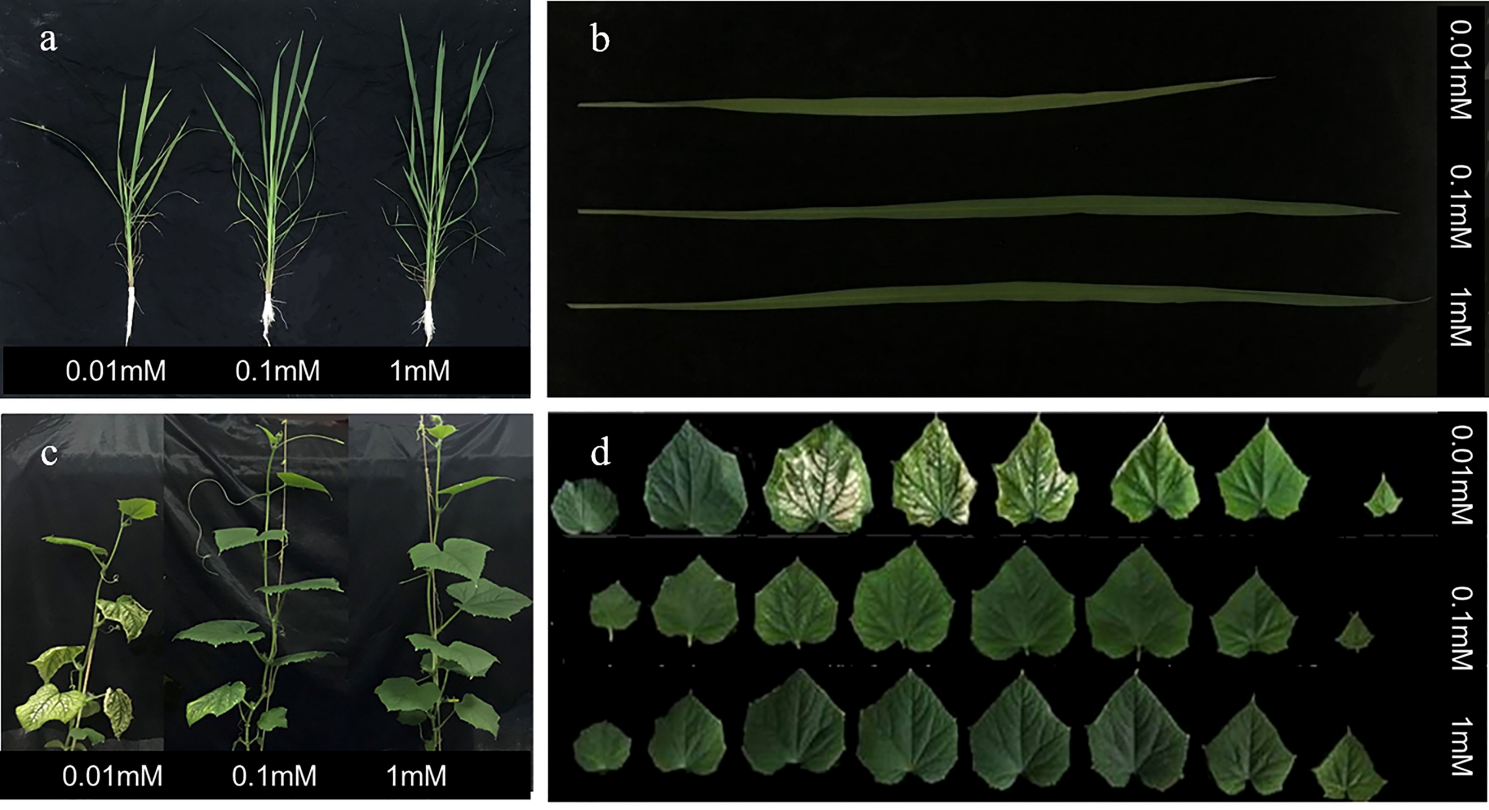
Morphological aspect of rice **(A, B)** and cucumber **(C, D)** under nutrient solution conditions at 0.01, 0.1, and 1 mM of Mg; rice and cucumber were captured on 25 and 15 days after the onset of treatment.

Rice and cucumber plants under 0.01 mM of Mg were seriously inhibited physiologically (**Figures 1A, C**). Cucumber developed typical Mg-deficient symptoms (interveinal chlorotic areas) on the middle leaves, and in magnesium-deficient rice plants, the leaf area was inhibited, while leaf chlorosis was not observed (**Figures 1B, D**).

Most of the physiological traits of rice and cucumber declined as Mg availability was downregulated, and cucumber plants were more sensitive than rice plants (**Table 1**). Mg concentration was the most affected trait, with an 88.6% and 72.4% decrease in rice and cucumber, respectively, at 0.01 mM of Mg compared with 1 mM of Mg. Cucumber chlorophyll and carotenoid contents (Chl and Car) significantly decreased even at 0.1 mM of Mg (**Table 1**). Compared with the 1-mM Mg treatment, rice and cucumber Chl decreased by 28.8% and 59.5%, respectively, under the 0.01-mM treatment. Mg deficiency significantly decreased cucumber Car and increased cucumber LMA, while no significant difference was observed in rice Car and LMA (**Table 1**).

**Table 1:** Effects of Mg supply on the morphological and physiological traits of rice and cucumber.

Species	Treatment	Biomass (g plant^−1^)	Area (cm^2^ plant^−1^)	LMA (g m^−2^)	Chl (mg g^−1^ FW)	Car (mg g^−1^ FW)	Mg concentration (mg g^−1^)
Rice	0.01 mM	1.46 ± 0.16b	354 ± 43b	28.4 ± 1.8a	2.15 ± 0.17b	0.36 ± 0.03a	0.93 ± 0.05c
	0.1 mM	1.67 ± 0.32ab	427 ± 66ab	27.7 ± 0.9a	2.65 ± 0.15a	0.39 ± 0.02a	4.40 ± 0.10b
	1 mM	1.80 ± 0.18a	445 ± 43a	27.9 ± 3.5a	2.86 ± 0.07a	0.38 ± 0.02a	8.10 ± 0.50a
Cucumber	0.01 mM	0.88 ± 0.0 b	322 ± 51b	20.7 ± 2.6b	0.79 ± 0.13c	0.15 ± 0.02c	1.41 ± 0.35c
	0.1 mM	1.05 ± 0.10a	353 ± 66ab	17.1 ± 1.3a	1.69 ± 0.14b	0.30 ± 0.02b	2.07 ± 0.23b
	1 mM	1.15 ± 0.13a	434 ± 72a	15.1 ± 1.2a	1.95 ± 0.11a	0.33 ± 0.02a	5.10 ± 0.41a

Data represent mean ± standard deviation (SD) of four replicates for biomass, area, LMA, Chl, Car, and Mg concentration. Different letters indicate statistically significant differences (P< 0.05) between three Mg treatments.

Area, leaf area; LMA, leaf mass per area; Chl, chlorophyll content; Car, carotenoid content.

### Photosynthesis response to irradiation and CO_2_ concentration

Rice and cucumber *P*_n_ declined gradually under increased Mg-deficient conditions (**Table 2**). Plants of both species grown in 0.01 mM of Mg exhibited 52.8% and 53.1% lower *P*_n_ than in 1 mM of Mg. Other photosynthetic parameters (*g*_s_, *g*_m_, and *C*_c_) were also limited with increasing Mg deficiency severity, while for *C*_i_, no significant difference was observed in 0.01 mM, compared with 1 mM Mg treatments (**Table 2**). Cucumber was more sensitive than rice under mild Mg deficiency (0.1 mM of Mg), while photosynthetic response under severe Mg deficiency (0.01 mM of Mg) was the same for both species.

**Table 2:** Effects of Mg supply on photosynthetic parameters of rice and cucumber.

Species	Treatment	*P*_n_ (μmol m^−2^ s^−1^)	*g*_s_ (mol·m^−2^·s^−1^)	*g*_m_ (mol m^−2^ s^−1^)	*C*_i_ (μmol mol^−1^)	*C*_c_ (μmol mol^−1^)
Rice	0.01 mM	11.5 ± 0.7c	0.236 ± 0.046b	0.072 ± 0.002c	304 ± 15a	143 ± 18b
	0.1 mM	20.7 ± 0.8b	0.521 ± 0.048a	0.157 ± 0.007b	315 ± 8a	183 ± 7a
	1 mM	24.4 ± 1.7a	0.552 ± 0.078a	0.200 ± 0.014a	308 ± 7a	186 ± 10a
Cucumber	0.01 mM	8.4 ± 0.9c	0.214 ± 0.039b	0.048 ± 0.008c	318 ± 11a	143 ± 8b
	0.1 mM	15.2 ± 1.8b	0.292 ± 0.080b	0.110 ± 0.005b	290 ± 18b	152 ± 3b
	1 mM	17.9 ± 0.9a	0.432 ± 0.033a	0.130 ± 0.009a	311 ± 7ab	173 ± 8a
Rice		***	**	***	ns	**
Cucumber		**	**	**	*	***

Data represent mean ± standard deviation (SD) of four replicates for *P*_n_, *g*_s_, *g*_m_, *C*_i_, and *C*_c_. Different letters indicate statistically significant differences (P< 0.05) between three Mg treatments. In the last two lines, the significance of each correlation between Mg concentration and photosynthetic parameters was represented by asterisks: *P< 0.05; **P< 0.01; ***P< 0.001; ns, no significant difference.

*P*_n_, net photosynthetic rate; *g*_s_, stomatal conductance; *g*_m_, mesophyll conductance; C_i_, intercellular CO_2_ concentration; *C*_c_, CO_2_ concentration in chloroplasts.

**Figure 2:**
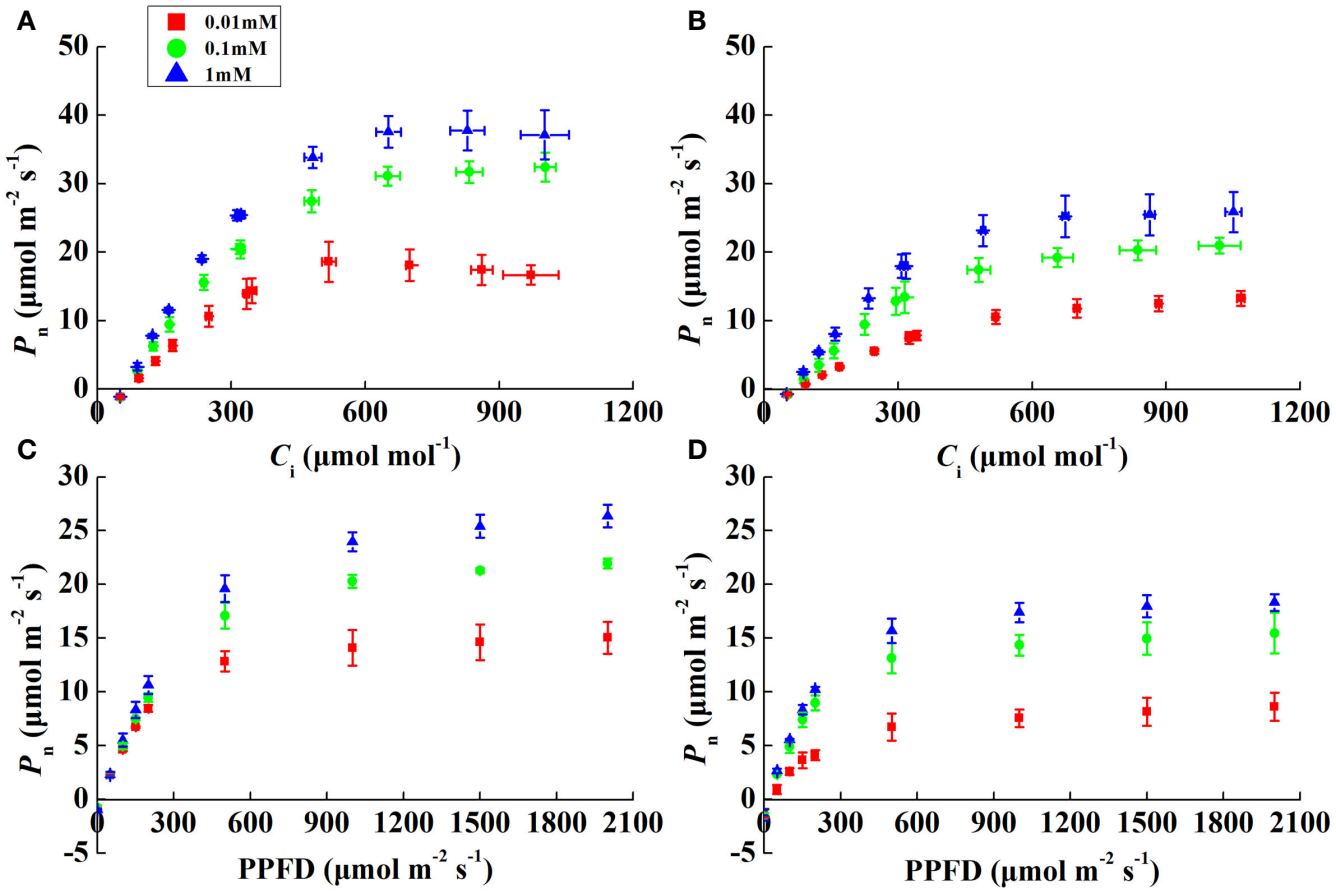
CO_2_ **(A, B)** and light **(C, D)** response curves showing the net photosynthetic rate (*P*_n_) in rice and cucumber leaves under Mg-deficient (0.01, 0.1 mM) and sufficient Mg (1 mM) supply treatments. Values represent mean ± standard deviation (SD) of three replicates.

*P*_n_ increased rapidly, peaked, and then stabilized with increasing irradiance and CO_2_ concentrations (**Figures 2A–D**). The *α* of both species was alike except for 0.01 mM Mg treatment, where it decreased by 20.7% and 53.4% in rice and cucumber, respectively, under 0.01 mM of Mg compared with 1 mM of Mg (**Table 3**). Despite *α*, other rice and cucumber biochemical parameters (CE, *V*_cmax_, and *J*_max_) significantly decreased under Mg deficiency and were consistent among the two plant species. Cucumber was more sensitive to Mg deficiency than rice, with wider disparities in *J*_max_/*V*_cmax_ and *C*_trans_ between 0.1 and 1 mM Mg treatments.

**Table 3:** Effects of Mg supply on photosynthetic parameters of light and CO_2_ response curves.

Species	Treatment	*α*	CE	*V*_cmax_ (μmol m^−2^ s^−1^)	*J*_max_ (μmol m^−2^ s^−1^)	*J*_max_/*V*_cmax_	*C*_trans_ (μmol mol^−1^)
Rice	0.01 mM	0.046 ± 0.001b	0.064 ± 0.007c	53.0 ± 7.0b	88 ± 12c	1.65 ± 0.01b	432 ± 3b
	0.1 mM	0.051 ± 0.001b	0.096 ± 0.007b	85.3 ± 8.4a	154 ± 9b	1.81 ± 0.09a	505 ± 46a
	1 mM	0.058 ± 0.005a	0.118 ± 0.003a	99.4 ± 6.4a	180 ± 15a	1.81 ± 0.03a	508 ± 17a
Cucumber	0.01 mM	0.027 ± 0.005c	0.034 ± 0.004c	38.3 ± 2.1c	63 ± 6c	1.64 ± 0.04b	426 ± 16b
	0.1 mM	0.051 ± 0.000b	0.060 ± 0.006b	58.9 ± 2.0b	99 ± 5b	1.69 ± 0.04b	446 ± 17b
	1 mM	0.058 ± 0.004a	0.081 ± 0.003a	69.3 ± 5.7a	122 ± 13a	1.76 ± 0.03a	481 ± 15a
Rice		**	***	***	***	*	*
Cucumber		*	**	**	**	**	**

Data represent mean ± standard deviation (SD) of three replicates for α, CE, *V*_cmax_, *J*_max_, *J*_max_/*V*_cmax_, and *C*_trans_. Different letters indicate statistically significant differences (P< 0.05) between three Mg treatments. In the last two lines, the significance of each correlation between Mg concentration and photosynthetic parameters was represented by asterisks: *P< 0.05; **P< 0.01; ***P< 0.001; ns, no significant difference.

α, apparent quantum yield; CE, carboxylation rate; *V*_cmax_, the maximum Rubisco carboxylation rate; *J*_max_, the maximum rate of electron transport; *C*_trans_, CO_2_ concentration in chloroplasts at which the transition from Rubisco to RuBP regeneration limitation occurs.

*J* and ΦPSII significantly declined with Mg deficiency regardless of plant species (**Table 4**). *F*_v_/*F*_m_ was significantly affected only in cucumber under 0.01 mM Mg treatment, where it decreased by 11.5%. Additionally, rice and cucumber plant NPQ under severe Mg-deficient treatment (0.01 mM of Mg) increased by 14.0% and decreased by 37.8%, respectively. There was no difference in rice α_leaf_ × β between Mg-deficient and Mg-sufficient leaves, while cucumber α_leaf_ × β decreased by 18.4% in 0.01 mM Mg treatment. For ΦNPQ, rice and cucumber showed different responses to Mg deficiency, while cucumber Φf,D increased by 40.4% under 0.01 mM Mg treatment. *J*_excess_ declined significantly only in rice under Mg deficiency, and rice and cucumber plant *J*_excess_/*J* under 0.01 mM of Mg increased by 27.9% and 39.9%, respectively.

**Table 4:** Effects of Mg supply on chlorophyll fluorescence parameters of rice and cucumber.

Species	Treatment	*J* (μmol m^−2^ s^−1^)	*F*_v_/*F*_m_	NPQ	*α*_leaf_ × *β*	ΦPSII	ΦNPQ	Φf,D	*J*_excess_ (μmol m^−2^ s^−1^)	*J*_excess_/*J* (%)
Rice	0.01 mM	97 ± 12c	0.802 ± 0.003a	1.788 ± 0.084a	0.483 ± 0.024a	0.159 ± 0.028c	0.539 ± 0.012a	0.302 ± 0.018ab	50 ± 9b	51 ± 3a
	0.1 mM	140 ± 6b	0.800 ± 0.009a	1.490 ± 0.109b	0.484 ± 0.020a	0.220 ± 0.009b	0.466 ± 0.017b	0.313 ± 0.013a	56 ± 3b	40 ± 1b
	1 mM	171 ± 12a	0.808 ± 0.001a	1.569 ± 0.113b	0.490 ± 0.029a	0.261 ± 0.012a	0.451 ± 0.020b	0.288 ± 0.009b	68 ± 6a	40 ± 2b
Cucumber	0.01 mM	84 ± 7b	0.707 ± 0.019b	0.952 ± 0.056b	0.413 ± 0.042b	0.132 ± 0.012b	0.423 ± 0.010b	0.445 ± 0.018a	49 ± 5a	58 ± 2a
	0.1 mM	117 ± 14a	0.796 ± 0.003a	1.464 ± 0.080a	0.491 ± 0.040a	0.183 ± 0.021a	0.485 ± 0.022a	0.332 ± 0.007b	53 ± 7a	46 ± 1b
	1 mM	126 ± 10a	0.799 ± 0.002a	1.531 ± 0.051a	0.506 ± 0.018a	0.198 ± 0.016a	0.485 ± 0.011a	0.317 ± 0.009b	52 ± 8a	41 ± 3c

Data represent mean ± standard deviation (SD) of three replicates for *J*, *F*_v_/*F*_m_, NPQ, α_leaf_ × β, ΦPSII, ΦNPQ, Φf,D, *J*_excess_, and *J*_excess_/*J*. Different letters indicate statistically significant differences (P< 0.05) between three Mg treatments.

*J*, electron transport rate; *F*_v_/*F*_m_, the maximal quantum efﬁciency of photosystem II; NPQ, non-photochemical quenching; α_leaf_: the leaf absorbance; β, the partitioning of the absorbed quanta between photosystems II and I; ΦPSII, the effective quantum yield of photosystem II; ΦNPQ, the proportion of thermally dissipated energy through NPQ; Φf,D, the fraction of absorbed light energy dissipated by additional quenching mechanism; *J*_excess_, the excess of photosynthetic linear electron transport not used for carbon assimilation.

Rice photorespiration rate (*P*_r_) significantly increased in 0.01 mM Mg treatment, by 42.9% compared with Mg-sufficient treatment. However, the highest cucumber *P*_r_ was found in 0.1 mM Mg treatment, where it increased by 14.3% compared with Mg-sufficient treatment. Compared with 1 mM Mg treatment, rice and cucumber *P*_r_/*P*_n_ (2%O_2_, the net photosynthetic rate in 2%O_2_) ratios all increased as the Mg supply was reduced, by 71.8% and 56.8%, respectively, at 0.01 mM of Mg (**Table 5**). In comparison with the 1-mM Mg treatment, rice and cucumber Γ* increased by 11.9% and 16.9%, respectively, under severe Mg deficiency (0.01 mM of Mg).

**Table 5:** Effects of Mg supply on photorespiration parameters of rice and cucumber.

Species	Treatment	*P*_n_ (μmol m^−2^ s^−1^)	*P*_n_2%O_2_ (μmol m^−2^ s^−1^)	*P*_r_ (μmol m^−2^ s^−1^)	*P*_r_/*P*_n_2%O_2_ (%)	Γ* (μmol mol^−1^)
Rice	0.01 mM	14.7 ± 1.6b	19.7 ± 1.9b	5.0 ± 0.4a	25.6 ± 0.6a	37.7 ± 0.3a
	0.1 mM	16.3 ± 2.3b	20.5 ± 3.1b	4.4 ± 1.0ab	20.1 ± 3.2b	33.9 ± 0.9b
	1 mM	20.2 ± 0.7a	23.7 ± 0.7a	3.5 ± 0.4b	14.9 ± 1.6c	33.7 ± 0.6b
Cucumber	0.01 mM	9.1 ± 1.3c	13.2 ± 1.2c	4.1 ± 0.2b	31.2 ± 3.4a	40.8 ± 1.6a
	0.1 mM	12.0 ± 1.0b	16.8 ± 0.9b	4.8 ± 0.1a	28.6 ± 2.1a	35.7 ± 0.6b
	1 mM	17.0 ± 0.6a	21.3 ± 0.3a	4.2 ± 0.3b	19.9 ± 1.6b	34.9 ± 2.5b

Data represent mean ± standard deviation (SD) of three replicates for *P*_n_, *P*_n_2%O_2_, *P*_r_, *P*_r_/*P*_n_2%O_2_, and Γ*. Different letters indicate statistically significant differences (P< 0.05) between three Mg treatments.

*P*_n_, net photosynthetic rate; *P*_n_2%O_2_, net photosynthetic rate in 2%O_2_; *P*_r_, photorespiration rate; Γ*, the CO_2_ compensation point in the absence of mitochondrial respiration.

### Dynamic photosynthesis changes

**Figure 3:**
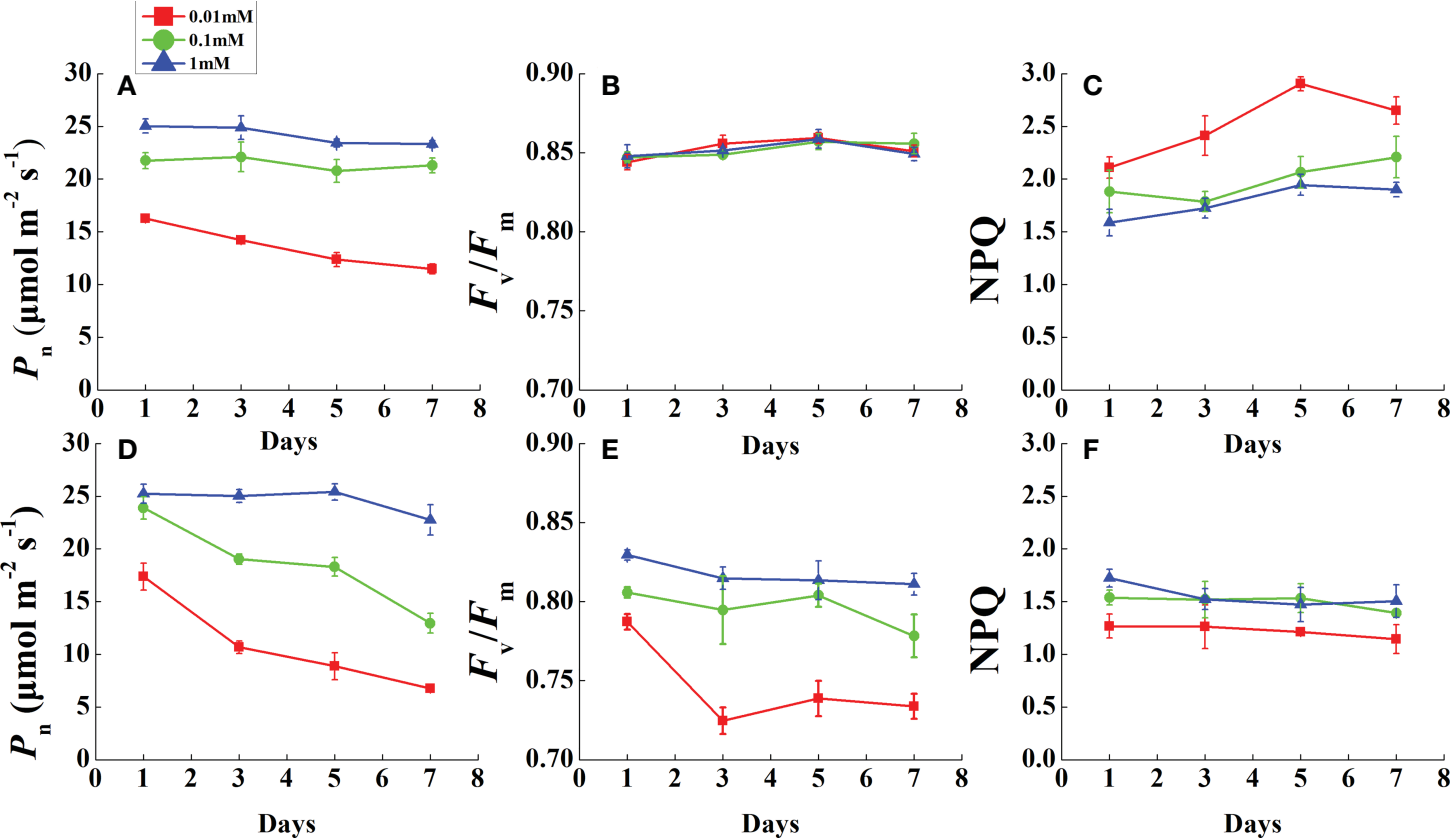
Variation of photosynthesis and chlorophyll fluorescence parameters in fully expanded leaves of rice **(A–C)** and cucumber **(D–F)** under different Mg supply treatments during 7 days. Values represent mean ± standard deviation (SD) of four replicates. *P*_n_, net photosynthetic rate; *F*_v_/*F*_m_, the maximal quantum efficiency; NPQ, non-photochemical quenching.

During the monitoring period, *P*_n_ was restricted prior to leaf necrosis under magnesium deficiency (**Figures 3A, D**). After the first measurement in 1 day, the *P*_n_ of all Mg-deficient treatments (except 0.1 mM of Mg in rice) decreased rapidly and continued to decline until the end of the measurements. As *P*_n_ declined, *F*_v_/*F*_m_ of all rice treatments was stable and unchanged (**Figure 3B**). However, cucumber *F*_v_/*F*_m_ in 0.01 mM Mg treatment was downregulated dramatically after 1 day and stabilized at a low level (**Figure 3E**). Mg deficiency had opposite effects on rice and cucumber NPQ during this period (**Figures 3C, F**). Under 0.01 mM Mg condition, rice NPQ was significantly enhanced, while for cucumber, it significantly decreased. However, under 0.1 mM Mg condition, rice had a slight increment, while cucumber showed no significant difference compared with Mg-sufficient treatment.

### Rice and cucumber pigment composition

**Figure 4:**
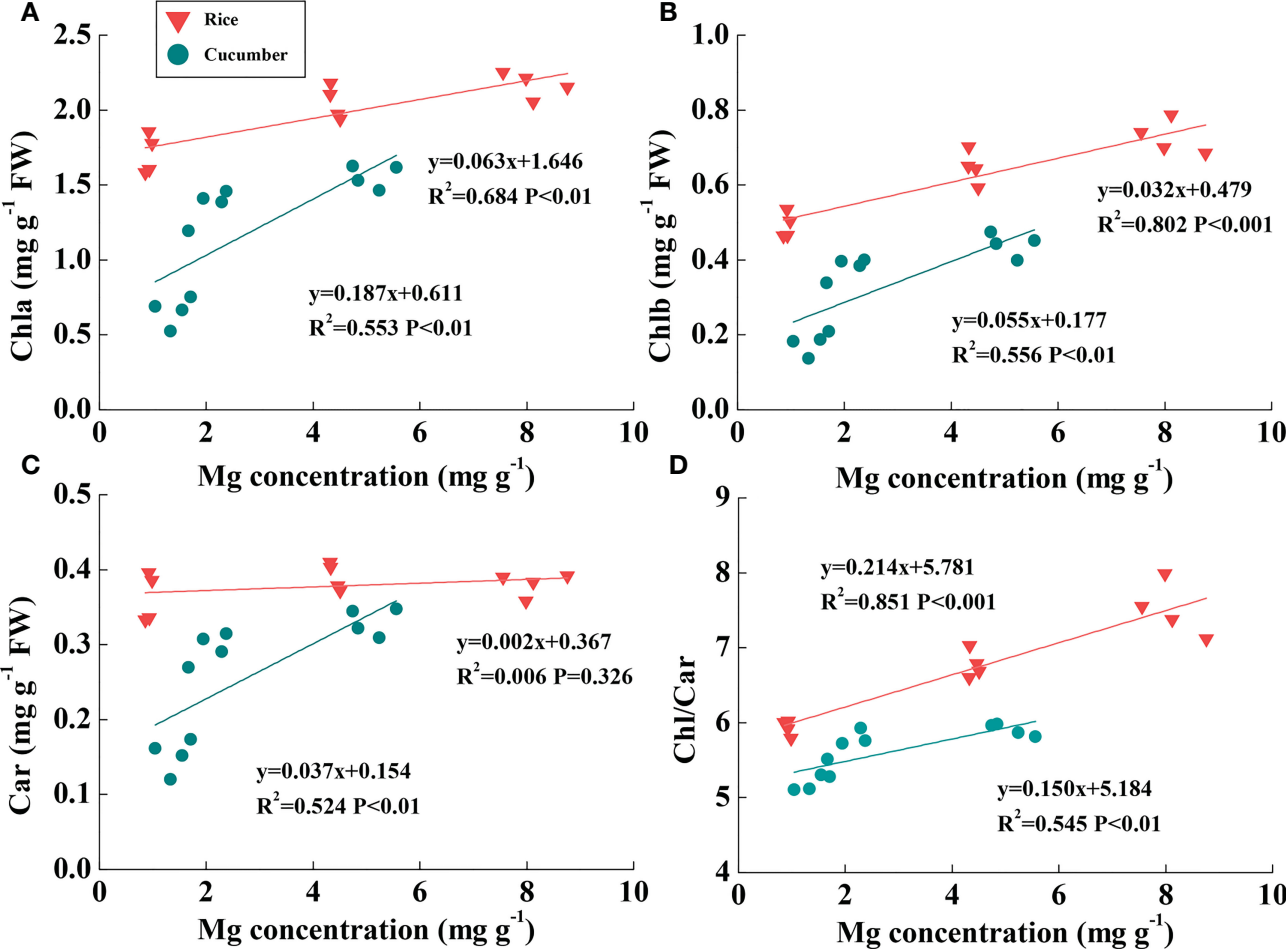
The correlations of Mg concentration with Chla **(A)**, Chlb **(B)**, Car **(C)**, and Chl/Car ratio **(D)** in the leaves of rice and cucumber. The lines represent the regressions that are best fit for the data (*n* = 12). Chla, chlorophyll a content; Chlb, chlorophyll b content; Chl, chlorophyll content; Car, carotenoid content.

Rice Mg concentration was higher than cucumber under the same Mg treatment; additionally, rice leaf pigment contents were much greater than those of cucumber (**Figures 4A–C**). Linear relationships between chlorophyll content and leaf Mg concentration were found across Mg treatments of both plant species (**Figures 4A, B**). Chla and Chlb were both positively correlated with rice and cucumber leaf Mg concentrations. Large slope variations between the two plant species showed that cucumber chlorophylls were more sensitive than those of rice in response to changing leaf Mg conditions. Car was positively correlated with leaf Mg concentration in cucumber, while the correlation was not found in rice (**Figure 4C**). Additionally, Chl/Car positively correlated with Mg concentration in rice and cucumber, but rice exhibited a more sensitive response to Mg deficiency compared with cucumber (**Figure 4D**).

## Discussion

### Mg deficiency restrains rice and cucumber growth

Decreased biomass is a universal phenomenon in Mg-deficient plants (Mengutay et al., 2013; Trankner et al., 2016). Rice and cucumber biomass was significantly reduced under low Mg concentration (0.01 mM) (**Table 1**). The two plant species exhibited a similar response to Mg deficiency, with a decrease in biomass and leaf area (**Table 1**), in accordance with previous observations (Chen and Fan, 2018; Peng et al., 2019; Ye et al., 2019). For LMA, different responses to Mg deficiency were observed among species. However, it obviously increased only in cucumber, which could be attributed to relative carbon accumulation (Trankner and Jaghdani, 2019).

Under Mg deficiency, cucumber was more sensitive than rice, with a more marked decline in Chl and Car (**Table 1**, **Figure 4**). Considering the more restricted Mg concentration and relatively high Chl in rice than in cucumber, rice effectively utilized Mg under Mg deficiency. In addition to Chl, Car also acts as a constituent of the light-harvesting antenna system in photosynthetic organisms (Ashraf and Harris, 2013). Importantly, Car can quench triplet chlorophyll and scavenge ROS like single oxygen which damages membranes and dissipates excess energy *via* xanthophyll-mediated NPQ (Cazzonelli, 2011). Consistent with our cucumber data, in the dicot *Sulla carnosa*, total Car decreased by 37.2% under extreme Mg-deficient conditions compared with Mg-sufficient plants (Farhat et al., 2015). Thus, photosynthesis-related antenna pigments were damaged by Mg deficiency stress, resulting in reduced light-harvesting efficiency, which affected cucumber more than rice.

### Mg deficiency restrains rice and cucumber photosynthesis

An Mg-deficiency-induced lower photosynthetic rate has been shown in rice, barley, and citrus plants (Yang et al., 2012; Trankner et al., 2016; Li et al., 2020). In our study, both rice and cucumber photosynthetic rates were downregulated under Mg deficiency (**Table 2**), coinciding with significant *g*_s_ and *g*_m_ decreases and the same response to Mg deficiency. However, a low *g*_s_ might not be the major factor driving photosynthesis decline under Mg-deficient stress, because rice and cucumber *C*_i_ at 0.01 mM of Mg was no different than at 1 mM of Mg (**Table 2**). Importantly, *g*_m_ plays a vital role in transmitting CO_2_ from the substomata to chloroplasts, and considering its decline accompanied by downregulated *C*_c_ in the two plant species, we speculate that it might be an essential cause of the reduced photosynthetic rate of Mg-deficient plants. Generally, *g*_m_ is related to leaf anatomic structure, aquaporin characteristics on membranes, and carbonic anhydrase activity in the cytosol and chloroplasts (Evans et al., 2009; Gao et al., 2010; Hu et al., 2012). The inherent declining *g*_m_ mechanism under Mg deficiency deserves further study.

Notably, *α* and CE decreased significantly under Mg-deficient treatments (**Table 3**), which implies impaired light-harvesting and carboxylation of both rice and cucumber. Significantly decreased *α* in cucumber under severe Mg-deficient treatment suggests that its light-harvesting system suffered more damage than that in rice. Under low Mg supply, rice and cucumber *J*_max_ and *V*_cmax_ declined to different degrees, with rice exhibiting more tolerance to Mg-deficient stress than cucumber (**Table 3**). Mg acts as an activator of the Calvin–Benson cycle enzymes (Schneider et al., 1992), and the declining *V*_cmax_ might be attributed to lower Mg^2+^ in chloroplasts (**Table 3**). In rice and cucumber, *J*_max_ and *V*_cmax_ displayed the same response to Mg deficiency, and similarly, *J*_max_ limitation in photosynthesis is also apparent in Mg-deficient grapevines (Rogiers et al., 2020). Considering the downregulation of these two vital biochemical parameters, severe CO_2_ utilization limitation may occur in Mg-deficient plants. Rice and cucumber all exhibited downregulated *J*_max_/*V*_cmax_ under 0.01 mM of Mg, while cucumber *J*_max_/*V*_cmax_ downregulated even under slight Mg deficiency (0.1 mM). Furthermore, the declining *J*_max_/*V*_cmax_ and *C*_trans_ suggest a greater limitation by RuBP regeneration than by carboxylation, which implies that under Mg deficiency, the limited energy generated by the electron transport was unable to meet the normal demands for CO_2_ fixation (Yamori et al., 2011). The 0.1 mM Mg treatment significantly decreased cucumber *C*_trans_ (**Table 3**), showing that cucumber was more restricted than rice in terms of *J*_max_ rather than *V*_cmax_.

Rice and cucumber ΦPS(II) and *J* all decreased as the Mg supply reduced (**Table 4**), implying declined PSII activity and damaged electron transfer components. Under low Mg supply, ΦPS(II) and *J* were also affected in sugar beet and *S. carnosa* (Hermans et al., 2004; Farhat et al., 2015). Strengthened photorespiration in low Mg supply suggests that the declined *J* may be attributed to the distribution of electrons to photorespiration or other alternative electron sinks and, alternatively, that the electron transport pool to carboxylation is reduced (Yiotis and Manetas, 2010).

Interestingly, only cucumber *F*_v_/*F*_m_ significantly decreased under Mg deficiency (**Table 4**, **Figures 3B, E**), which implies that rice, despite being exposed to the same stress, did not suffer from photoinhibition. *F*_v_/*F*_m_ downregulation has been reported in Mg-deficient *S. carnosa* (Farhat et al., 2015), *Vicia faba* (Hariadi and Shabala, 2004), and *Citrus* seedlings (Yang et al., 2012). Other studies have shown that fluorescence parameters including *F*_v_/*F*_m_ are not affected in rice and *Helianthus annuus* (Lasa et al., 2000; Li et al., 2020), and also verified our result that rice was more tolerant than cucumber to Mg deficiency. NPQ is the major mechanism to avoid photoinhibition, whereby plants convert excess light energy into heat energy (Muller et al., 2001). Elevated NPQ occurs in plants under stress; notably, it increased in rice but decreased in cucumber. Analogous to *F*_v_/*F*_m_, the irregular variation in NPQ might be species-dependent. Additionally, NPQ plays a crucial role in regulating PSII activity under Mg deficiency, and elevated NPQ is always accompanied by excessive ATP and accumulated H^+^ in the thylakoid stroma (Kulheim et al., 2002).

### Different Mg deficiency sensitivities correlate with unique photoprotective performance

Leaves become chlorotic under Mg deficiency as a consequence of photobleaching; therefore, plants will enhance key ROS-scavenging enzyme activities to remove photooxidation damage (Kanazawa et al., 2000). Aside from ROS scavenging systems, plants strengthen photorespiration and NPQ to consume excess light energy (Kozaki and Takeba, 1996; Muller et al., 2001). We compared rice and cucumber photorespiration performance and found that rice exhibited a more marked rise in *P*_r_ under Mg deficiency. However, compared with Mg-sufficient treatment, increasing *P*_r_/*P*_n_ (2%O_2_) ratio rates were similar between rice and cucumber under Mg deficiency treatments (**Table 5**). Γ* can be used to evaluate photorespiration rates indirectly (Busch, 2013), and elevated levels have been observed in plants under high temperatures or excessive nitrogen supply (Brooks and Farquhar, 1985; Li et al., 2009). Our results further demonstrate that increased Γ* strengthened photorespiration competence in rice and cucumber under Mg deficiency (**Table 5**). Unexpectedly, rice photorespiration-related parameters were analogous to those of cucumber. Therefore, the stronger photoprotection capacity of rice compared with cucumber cannot be attributed to higher photorespiration.

A 7-day-long dynamic monitoring was conducted before cucumber leaf chlorosis. During this time, Mg-deficient rice leaves all remained green, while cucumber leaves became chlorotic. Interestingly, in rice, *F*_v_/*F*_m_ was maintained even as *P*_n_ was downregulated under Mg deficiency (**Figures 3A, B**). Under stress, NPQ is expected to increase, but cucumber NPQ decreases under Mg-deficient stress (**Figure 3F**). Considering that rice photosynthetic gas exchange and photorespiration-related parameters were all analogous to those of cucumber, NPQ may be an important factor in determining their differing responses to Mg deficiency.

We suggest that the destroyed chloroplast ultrastructure during leaf chlorosis might affect NPQ function. However, Mg-deficient cucumber NPQ declined on the first day before the leaf became chlorotic; therefore, the decline might be attributed to other factors. In a study of *Abies alba* MILL photoprotection (Doerken and Lepetit, 2018), sun leaves were exposed to high light radiation and did not suffer from more photoinhibition than shade leaves. Compared with Mg-deficient rice and cucumber, *Abies* sun leaves exhibited much higher NPQ and more than twice as much xanthophyll per total chlorophyll as the shade leaves. The xanthophyll cycle-dependent NPQ is a major mechanism to avoid photoinhibition (Demmig-Adams and Adams, 2006). NPQ is positively correlated with zeaxanthin and antheraxanthin contents (Niyogi et al., 1997), and xanthophyll cycle pigments are carotenoids. For cucumber, a positive linear relationship was found between Car and Mg concentration (**Figure 4C**). However, there was no direct evidence that Mg deficiency lowered xanthophyll cycle pigment concentrations. Thus, a further study determining Car composition should be conducted to investigate the mechanism underlying decreased NPQ under Mg deficiency.

Cucumber Φf,D significantly increased as ΦNPQ decreased under Mg deficiency, while rice Φf,D was relatively constant (**Table 4**), indicating that low cucumber NPQ under Mg deficiency may induce light excitation dissipation by additional quenching mechanisms. After calculating *J*_excess_, we found that cucumber *J*_excess_/*J* exposed to the lowest Mg supply (0.01 mM) increased by 39.9%, while in rice, it was only 27.9% under the same conditions. It was reported that high rates of electron transfer to some additional/alternative electron transport pathways, such as PTOX-mediated electron transport to oxygen and Mehler reaction, may dissipate excess electrons (Asada, 1999; Cournac et al., 2000). PTOX can transfer electrons from plastoquinone to oxygen without generating ROS; normally, it is only a minor protein and its capacity for consumption of excess electrons appears to be low (Ort and Baker, 2002; Peltier and Cournac, 2002; Josse et al., 2003). However, it has been demonstrated that PTOX-mediated electron transport to oxygen is greatly upregulated and may play a critical role in preventing over-reduction of the photosynthetic electron transport chain under various stress conditions (McDonald et al., 2011). Considering that rice photorespiration-related parameters are analogous to those of cucumber, it is clear that PTOX-mediated electron flow to oxygen might be the most probable alternative electron flow involved in cucumber plant response to Mg deficiency. Furthermore, excess electrons are transported to oxygen in the Mehler reaction, which may form superoxide and finally increase oxidative stress (Huner et al., 1998). Meanwhile, in rice, ROS scavenging enzyme activities were relatively higher than in cucumber under different Mg levels (**Figure S1**). When comparing the correlations between Mg concentration and the Chl/Car ratio, rice exhibited a more rapid response to Mg deficiency than cucumber (**Figure 4D**). In considering the Car role in scavenging ROS, cucumber was equipped with lower Car under Mg deficiency and may have suffered more oxidative stress than rice.

**Figure 5:**
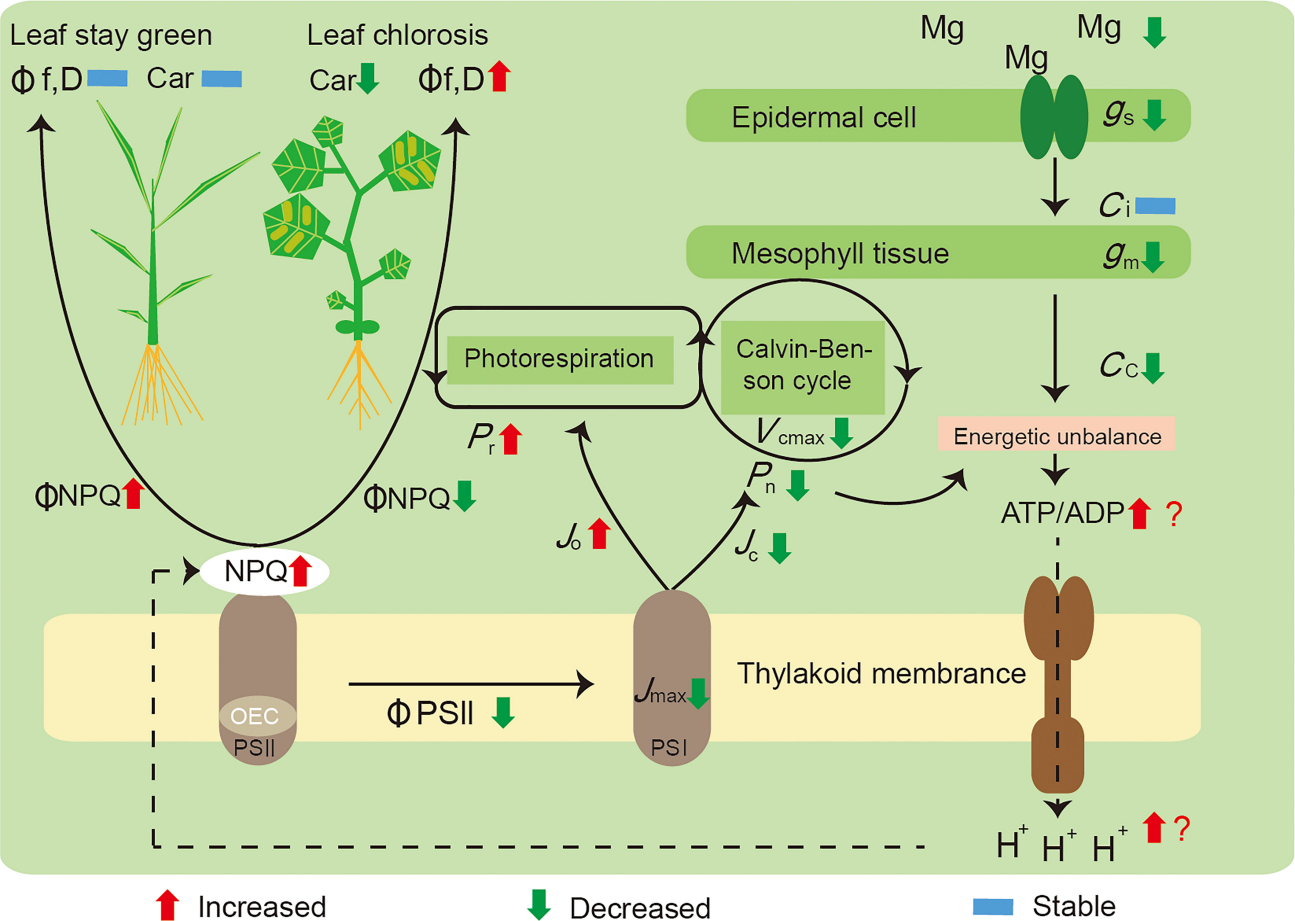
A model highlighting the main effects induced by Mg deficiency on the photosynthesis of rice and cucumber plants. Initially, a decreased concentration of Mg in the leaves of plants, influencing the Calvin–Benson cycle function through restricting *g*_m_ and *V*_cmax_, leading to a surplus of energy generated by photophosphorylation, with the ratio between ATP and ADP increased, yet more H^+^ accumulated and inducing NPQ increment; in parallel, Mg deficiency also increased the proportion of electron transport to photorespiration (*J*_o_), further competing with Rubisco carboxylation (*J*_c_). Mg-deficient cucumber showed a decreased ΦNPQ and a higher rate of electron transport to alternative pathways; thus, it suffered preferential damage than rice and became leaf chlorosis. *P*_n_, net photosynthetic rate; *P*_r_, photorespiration rate; *g*_s_, stomatal conductance; *g*_m_, mesophyll conductance; *C*_i_, intercellular CO_2_ concentration; *C*_c_, CO_2_ concentration in chloroplasts; *V*_cmax_, the maximum Rubisco carboxylation rate; *J*_max_, the maximum rate of electron transport; *J*_c_, electron flux to Rubisco carboxylation; *J*_o_, electron flux to Rubisco oxygenation; NPQ, non-photochemical quenching; OEC, oxygen-evolving complex; ΦPSII, the effective quantum yield of photosystem II; ΦNPQ, the proportion of thermally dissipated energy through NPQ; Φf,D, the fraction of absorbed light energy dissipated by additional quenching mechanism; PSI, photosystem I; PSII, photosystem II; Car, carotenoid content.

We summarized the mechanisms underlying these responses (**Figure 5**): i) a decreased Mg concentration in the plant leaves influenced the Calvin–Benson cycle function through restricting *g*_m_ and Rubisco activity (*V*_cmax_), which induced energy captured by light harvesting to exceed utilization by carbon assimilation; ii) Mg deficiency leads to a surplus of energy generated by photophosphorylation, with an increased ATP and ADP ratio, as well as more H^+^ accumulated in the thylakoid stroma inducing NPQ increment; and iii) in parallel, declining *J*_max_ and ΦPSII exhibited a RuBP regeneration limitation, and Mg deficiency also increased the proportion of electron transport to photorespiration (*J*_o_), further competing with Rubisco carboxylation (*J*_c_). Mg deficiency significantly decreased NPQ and increased electron transport rates to other oxygen-dependent pathways in cucumber; meanwhile, rice possessed a stronger photoprotection capacity than cucumber, was in a better condition, and remained green.

## Conclusions

Under magnesium deficiency stress, rice and cucumber growth and photosynthesis were inhibited. Suppressed photosynthesis was attributed to integrated limitations of *J*_max_, *V*_cmax_, and *g*_m_. Reduced photosynthetic rate provoked an imbalanced energy between light capture and utilization by carbon assimilation, inducing NPQ increment. In parallel, more electron transport to photorespiration further competed with Rubisco carboxylation. Greater cucumber sensitivity under Mg deficiency was attributed to lower NPQ and higher electron transport rates to alternative pathways and subsequently increased oxidative stress. Overall, these results revealed the inherent mechanism of suppressed photosynthesis and suggested the crucial role of photoprotection capacity under Mg deficiency.

## Data availability statement

The original contributions presented in the study are included in the article/**Supplementary Material**. Further inquiries can be directed to the corresponding author.

## Author contributions

SG, XM and SB conceived and designed the experiment. XM, SW and KC performed the experiments. XM, SB, YP and KX analyzed the data and were responsible for the table and figures. XM wrote the paper. SG, YP and MW proofread and polished the manuscript. All authors contributed to the article and approved the submitted version.

## Supplementary material

Supplementary Figure 1 Effects of Mg supply on the activities of SOD **(A)**, POD **(B)** and CAT **(C)** of rice and cucumber. Values represent mean ± standard deviation (SD) of four replicates.

## References

[B1] Asada K. (1999). The water-water cycle in chloroplasts: Scavenging of active oxygens and dissipation of excess photons. Annu. Rev. Plant Physiol. Plant Mol. Biol. 50, 601–639. doi: 10.1146/annurev.arplant.50.1.601 15012221

[B2] Ashraf M. Harris P. J. C. (2013). Photosynthesis under stressful environments: An overview. Photosynthetica 51, 163–190. doi: 10.1007/s11099-013-0021-6

[B3] Brooks A. Farquhar G. D. (1985). Effect of temperature on the CO_2_/O_2_ specificity of ribulose-1,5-bisphosphate carboxylase oxygenase and the rate of respiration in the light - estimates from gas-exchange measurements on spinach. Planta 165, 397–406. doi: 10.1007/BF00392238 24241146

[B4] Busch F. A. (2013). Current methods for estimating the rate of photorespiration in leaves. Plant Biol. 15, 648–655. doi: 10.1111/j.1438-8677.2012.00694.x 23186383

[B5] Cakmak I. Kirkby E. A. (2008). Role of magnesium in carbon partitioning and alleviating photooxidative damage. Physiologia Plantarum 133, 692–704. doi: 10.1111/j.1399-3054.2007.01042.x 18724409

[B6] Cazzonelli C. I. (2011). Carotenoids in nature: insights from plants and beyond. Funct. Plant Biol. 38, 833–847. doi: 10.1071/FP11192 32480941

[B7] Ceylan Y. Kutman U. B. Mengutay M. Cakmak I. (2016). Magnesium applications to growth medium and foliage affect the starch distribution, increase the grain size and improve the seed germination in wheat. Plant Soil 406, 145–156. doi: 10.1007/s11104-016-2871-8

[B8] Chen H. B. Fan X. L. (2018). Effects of magnesium remobilization and allocation on banana plant growth. J. Plant Nutr. 41, 1312–1320. doi: 10.1080/01904167.2018.1450422

[B9] Chen Z. C. Peng W. T. Li J. Liao H. (2018). Functional dissection and transport mechanism of magnesium in plants. Semin. Cell Dev. Biol. 74, 142–152. doi: 10.1016/j.semcdb.2017.08.005 28822768

[B10] Cournac L. Redding K. Ravenel J. Rumeau D. Josse E. M. Kuntz M.. (2000). Electron flow between photosystem II and oxygen in chloroplasts of photosystem I-deficient algae is mediated by a quinol oxidase involved in chlororespiration. J. Biol. Chem. 275, 17256–17262. doi: 10.1074/jbc.M908732199 10748104

[B11] Cowan J. A. (2002). Structural and catalytic chemistry of magnesium-dependent enzymes. Biometals 15, 225–235. doi: 10.1023/A:1016022730880 12206389

[B12] Demmig-Adams B. Adams W. W. (2002). Antioxidants in photosynthesis and human nutrition. Science 298, 2149–2153. doi: 10.1126/science.1078002 12481128

[B13] Demmig-Adams B. Adams W. W. (2006). Photoprotection in an ecological context: the remarkable complexity of thermal energy dissipation. New Phytol. 172, 11–21. doi: 10.1111/j.1469-8137.2006.01835.x 16945085

[B14] Ding Y. C. Chang C. R. Luo W. Wu Y. S. Ren X. L. Wang P.. (2008). High potassium aggravates the oxidative stress inducedy by magnesium deficiency in rice leaves. Pedosphere 18, 316–327. doi: 10.1016/S1002-0160(08)60021-1

[B15] Ding Y. Luo W. Xu G. (2006). Characterisation of magnesium nutrition and interaction of magnesium and potassium in rice. Ann. Appl. Biol. 149, 111–123. doi: 10.1111/j.1744-7348.2006.00080.x

[B16] Doerken V. M. Lepetit B. (2018). Morpho-anatomical and physiological differences between sun and shade leaves in Abies alba MILL. (Pinaceae, coniferales): a combined approach. Plant Cell Environ. 41, 1683–1697. doi: 10.1111/pce.13213 29664115

[B17] Evans J. R. Kaldenhoff R. Genty B. Terashima I. (2009). Resistances along the CO_2_ diffusion pathway inside leaves. J. Exp. Bot. 60, 2235–2248. doi: 10.1093/jxb/erp117 19395390

[B18] Farhat N. Elkhouni A. Zorrig W. Smaoui A. Abdelly C. Rabhi M. (2016). Effects of magnesium deficiency on photosynthesis and carbohydrate partitioning. Acta Physiologiae Plantarum 38, 10. doi: 10.1007/s11738-016-2165-z

[B19] Farhat N. Ivanov A. G. Krol M. Rabhi M. Smaoui A. Abdelly C.. (2015). Preferential damaging effects of limited magnesium bioavailability on photosystem I in Sulla carnosa plants. Planta 241, 1189–1206. doi: 10.1007/s00425-015-2248-x 25637102

[B20] Fischer E. S. Bremer E. (1993). Influence of magnesium-deficiency on rates of leaf expansion, starch and sucrose accumulation, and net assimilation in *Phaseolus*-*vulgaris* . Physiologia Plantarum 89, 271–276. doi: 10.1111/j.1399-3054.1993.tb00153.x

[B21] Gao Z. X. He X. L. Zhao B. C. Zhou C. J. Liang Y. Z. Ge R. C.. (2010). Overexpressing a putative aquaporin gene from wheat, TaNIP, enhances salt tolerance in transgenic arabidopsis. Plant Cell Physiol. 51, 767–775. doi: 10.1093/pcp/pcq036 20360019

[B22] Gransee A. Fuhrs H. (2013). Magnesium mobility in soils as a challenge for soil and plant analysis, magnesium fertilization and root uptake under adverse growth conditions. Plant Soil 368, 5–21. doi: 10.1007/s11104-012-1567-y

[B23] Hariadi Y. Shabala S. (2004). Screening broad beans (Vicia faba) for magnesium deficiency. II. Photosynthetic performance And leaf bioelectrical responses. Funct. Plant Biol. 31, 539–549. doi: 10.1071/FP03202 32688925

[B24] Harley P. C. Loreto F. Dimarco G. Sharkey T. D. (1992). Theoretical considerations when estimating the mesophyll conductance to CO_2_ flux by analysis of the response of photosynthesis to CO_2_ . Plant Physiol. 98, 1429–1436. doi: 10.1104/pp.98.4.1429 16668811 PMC1080368

[B25] Hauer-Jakli M. Trankner M. (2019). Critical leaf magnesium thresholds and the impact of magnesium on plant growth and photo-oxidative defense: A systematic review and meta-analysis from 70 years of research. Front. Plant Sci. 10, 15. doi: 10.3389/fpls.2019.00766 31275333 PMC6592071

[B26] Hendrickson L. Furbank R. T. Chow W. S. (2004). A simple alternative approach to assessing the fate of absorbed light energy using chlorophyll fluorescence. Photosynthesis Res. 82, 73–81. doi: 10.1023/B:PRES.0000040446.87305.f4 16228614

[B27] Hermans C. Johnson G. N. Strasser R. J. Verbruggen N. (2004). Physiological characterisation of magnesium deficiency in sugar beet: acclimation to low magnesium differentially affects photosystems I and II. Planta 220, 344–355. doi: 10.1007/s00425-004-1340-4 15378366

[B28] Hermans C. Vuylsteke M. Coppens F. Cristescu S. M. Harren F. J. M. Inze D.. (2010). Systems analysis of the responses to long-term magnesium deficiency and restoration in arabidopsis thaliana. New Phytol. 187, 132–144. doi: 10.1111/j.1469-8137.2010.03257.x 20412444

[B29] Hu W. Yuan Q. Q. Wang Y. Cai R. Deng X. M. Wang J.. (2012). Overexpression of a wheat aquaporin gene, TaAQP8, enhances salt stress tolerance in transgenic tobacco. Plant Cell Physiol. 53, 2127–2141. doi: 10.1093/pcp/pcs154 23161856

[B30] Huang Y. Jiao Y. Y. Nawaz M. A. Chen C. Liu L. Lu Z.. (2016). Improving magnesium uptake, photosynthesis and antioxidant enzyme activities of watermelon by grafting onto pumpkin rootstock under low magnesium. Plant Soil 409, 229–246. doi: 10.1007/s11104-016-2965-3

[B31] Huner N. P. A. Oquist G. Sarhan F. (1998). Energy balance and acclimation to light and cold. Trends Plant Sci. 3, 224–230. doi: 10.1016/S1360-1385(98)01248-5

[B32] Jansson S. (1994). The light-harvesting chlorophyll a/b binding-proteins. Biochim. Et Biophys. Acta-Bioenergetics 1184, 1–19. doi: 10.1016/0005-2728(94)90148-1 8305447

[B33] Josse E. M. Alcaraz J. P. Laboure A. M. Kuntz M. (2003). *In vitro* characterization of a plastid terminal oxidase (PTOX). Eur. J. Biochem. 270, 3787–3794. doi: 10.1046/j.1432-1033.2003.03766.x 12950262

[B34] Kanazawa S. Sano S. Koshiba T. Ushimaru T. (2000). Changes in antioxidative enzymes in cucumber cotyledons during natural senescence: comparison with those during dark-induced senescence. Physiologia Plantarum 109, 211–216. doi: 10.1034/j.1399-3054.2000.100214.x

[B35] Kozaki A. Takeba G. (1996). Photorespiration protects C3 plants from photooxidation. Nature 384, 557–560. doi: 10.1038/384557a0

[B36] Kulheim C. Agren J. Jansson S. (2002). Rapid regulation of light harvesting and plant fitness in the field. Science 297, 91–93. doi: 10.1126/science.1072359 12098696

[B37] Laing W. Greer D. Sun O. Beets P. Lowe A. Payn T. (2000). Physiological impacts of mg deficiency in pinus radiata: growth and photosynthesis. New Phytol. 146, 47–57. doi: 10.1046/j.1469-8137.2000.00616.x

[B38] Lasa B. Frechilla S. Aleu M. Gonzalez-Moro B. Lamsfus C. Aparicio-Tejo P. M. (2000). Effects of low and high levels of magnesium on the response of sunflower plants grown with ammonium and nitrate. Plant Soil 225, 167–174. doi: 10.1023/A:1026568329860

[B39] Li Y. Gao Y. X. Ding L. Shen Q. R. Guo S. W. (2009). Ammonium enhances the tolerance of rice seedlings (Oryza sativa l.) to drought condition. Agric. Water Manage. 96, 1746–1750. doi: 10.1016/j.agwat.2009.07.008

[B40] Li J. Yokosho K. Liu S. Cao H. R. Yamaji N. Zhu X. G.. (2020). Diel magnesium fluctuations in chloroplasts contribute to photosynthesis in rice. Nat. Plants 6, 848–84+. doi: 10.1038/s41477-020-0686-3 32541951

[B41] Lorimer G. H. Badger M. R. Andrews T. J. (1976). The activation of ribulose-1,5-bisphosphate carboxylase by carbon dioxide and magnesium ions. equilibria, kinetics, a suggested mechanism, and physiological implications. Biochemistry 15, 529–536. doi: 10.1021/bi00648a012 3199

[B42] Marschner H. (1995). Mineral nutrition of higher plants, second edition. Mineral Nutr. Higher Plants Second Edition. xv+889p.

[B43] McDonald A. E. Ivanov A. G. Bode R. Maxwell D. P. Rodermel S. R. Huenter N. P. A. (2011). Flexibility in photosynthetic electron transport: The physiological role of plastoquinol terminal oxidase (PTOX). Biochim. Et Biophys. Acta-Bioenergetics 1807, 954–967. doi: 10.1016/j.bbabio.2010.10.024 21056542

[B44] Mengutay M. Ceylan Y. Kutman U. B. Cakmak I. (2013). Adequate magnesium nutrition mitigates adverse effects of heat stress on maize and wheat. Plant Soil 368, 57–72. doi: 10.1007/s11104-013-1761-6

[B45] Muller P. Li X. P. Niyogi K. K. (2001). Non-photochemical quenching. a response to excess light energy. Plant Physiol. 125, 1558–1566. doi: 10.1104/pp.125.4.1558 11299337 PMC1539381

[B46] Niyogi K. K. Bjorkman O. Grossman A. R. (1997). Chlamydomonas xanthophyll cycle mutants identified by video imaging of chlorophyll fluorescence quenching. Plant Cell 9, 1369–1380. doi: 10.2307/3870388 12237386 PMC157004

[B47] Ort D. R. Baker N. R. (2002). A photoprotective role for O_2_ as an alternative electron sink in photosynthesis? Curr. Opin. Plant Biol. 5, 193–198. doi: 10.1016/S1369-5266(02)00259-5 11960735

[B48] Peltier G. Cournac L. (2002). Chlororespiration. Annu. Rev. Plant Biol. 53, 523–550. doi: 10.1146/annurev.arplant.53.100301.135242 12227339

[B49] Peng Y. Y. Liao L. L. Liu S. Nie M. M. Li J. Zhang L. D.. (2019). Magnesium deficiency triggers SGR-mediated chlorophyll degradation for magnesium remobilization. Plant Physiol. 181, 262–275. doi: 10.1104/pp.19.00610 31289214 PMC6716262

[B50] Rogiers S. Y. Greer D. H. Moroni F. J. Baby T. (2020). Potassium and magnesium mediate the light and CO_2_ photosynthetic responses of grapevines. Biology-Basel 9, 18. doi: 10.3390/biology9070144 32605293 PMC7407654

[B51] Savitch L. V. Ivanov A. G. Krol M. Sprott D. P. Oquist G. Huner N. P. A. (2010). Regulation of energy partitioning and alternative electron transport pathways during cold acclimation of lodgepole pine is oxygen dependent. Plant Cell Physiol. 51, 1555–1570. doi: 10.1093/pcp/pcq101 20630988

[B52] Schneider G. Lindqvist Y. Branden C. I. (1992). Rubisco - structure and mechanism. Annu. Rev. Biophysics Biomolecular Structure 21, 119–143. doi: 10.1146/annurev.bb.21.060192.001003 1525466

[B53] Sharkey T. D. Bernacchi C. J. Farquhar G. D. Singsaas E. L. (2007). Fitting photosynthetic carbon dioxide response curves for c-3 leaves. Plant Cell Environ. 30, 1035–1040. doi: 10.1111/j.1365-3040.2007.01710.x 17661745

[B54] Shaul O. (2002). Magnesium transport and function in plants: the tip of the iceberg. Biometals 15, 309–323. doi: 10.1023/A:1016091118585 12206396

[B55] Streb P. Josse E. M. Gallouet E. Baptist F. Kuntz M. Cornic G. (2005). Evidence for alternative electron sinks to photosynthetic carbon assimilation in the high mountain plant species ranunculus glacialis. Plant Cell Environ. 28, 1123–1135. doi: 10.1111/j.1365-3040.2005.01350.x

[B56] Traenkner M. Tavakol E. Jakli B. (2018). Functioning of potassium and magnesium in photosynthesis, photosynthate translocation and photoprotection. Physiologia Plantarum 163, 414–431. doi: 10.1111/ppl.12747 29667201

[B57] Trankner M. Jaghdani S. J. (2019). Minimum magnesium concentrations for photosynthetic efficiency in wheat and sunflower seedlings. Plant Physiol. Biochem. 144, 234–243. doi: 10.1016/j.plaphy.2019.09.040 31590092

[B58] Trankner M. Jakli B. Tavakol E. Geilfus C. M. Cakmak I. Dittert K.. (2016). Magnesium deficiency decreases biomass water-use efficiency and increases leaf water-use efficiency and oxidative stress in barley plants. Plant Soil 406, 409–423. doi: 10.1007/s11104-016-2886-1

[B59] Valentini R. Epron D. Deangelis P. Matteucci G. Dreyer E. (1995). *In-situ* estimation of net CO_2_ assimilation, photosynthetic electron flow and photorespiration in Turkey oak (Q-*cerris* l) leaves - diurnal cycles under different levels of water-supply. Plant Cell Environ. 18, 631–640. doi: 10.1111/j.1365-3040.1995.tb00564.x

[B60] Wang R. Huang J. Liang A. Wang Y. Mur L. A. J. Wang M.. (2020a). Zinc and copper enhance cucumber tolerance to fusaric acid by mediating its distribution and toxicity and modifying the antioxidant system. Int. J. Mol. Sci. 21 (9), 3370. doi: 10.3390/ijms21093370 32397623 PMC7247006

[B61] Wang Z. Ul Hassan M. Nadeem F. Wu L. Q. Zhang F. S. Li X. X. (2020b). Magnesium fertilization improves crop yield in most production systems: A meta-analysis. Front. Plant Sci. 10. doi: 10.3389/fpls.2019.01727 PMC6992656 32038691

[B62] White P. J. Broadley M. R. El-Serehy H. A. George T. S. Neugebauer K. (2018). Linear relationships between shoot magnesium and calcium concentrations among angiosperm species are associated with cell wall chemistry. Ann. Bot. 122, 221–226. doi: 10.1093/aob/mcy062 29722830 PMC6070070

[B63] Wilkinson S. R. Welch R. M. Mayland H. F. Grunes D. L. (1990). Magnesium in plants - uptake, distribution, function, and utilization by man and animals. Metal Ions Biol. Syst. 26, 33–56.

[B64] Wingler A. Brownhill E. Pourtau N. (2005). Mechanisms of the light-dependent induction of cell death in tobacco plants with delayed senescence. J. Exp. Bot. 56, 2897–2905. doi: 10.1093/jxb/eri284 16157651

[B65] Xie K. L. Cakmak I. Wang S. Y. Zhang F. S. Guo S. W. (2021). Synergistic and antagonistic interactions between potassium and magnesium in higher plants. Crop J. 9, 249–256. doi: 10.1016/j.cj.2020.10.005

[B66] Yamori W. Nagai T. Makino A. (2011). The rate-limiting step for CO_2_ assimilation at different temperatures is influenced by the leaf nitrogen content in several c-3 crop species. Plant Cell Environ. 34, 764–777. doi: 10.1111/j.1365-3040.2011.02280.x 21241332

[B67] Yang G. H. Yang L. T. Jiang H. X. Li Y. Wang P. Chen L. S. (2012). Physiological impacts of magnesium-deficiency in citrus seedlings: photosynthesis, antioxidant system and carbohydrates. Trees-Structure Funct. 26, 1237–1250. doi: 10.1007/s00468-012-0699-2

[B68] Ye X. Chen X. F. Deng C. L. Yang L. T. Lai N. W. Guo J. X.. (2019). Magnesium-deficiency effects on pigments, photosynthesis and photosynthetic electron transport of leaves, and nutrients of leaf blades and veins in citrus sinensis seedlings. Plants-Basel 8, 20. doi: 10.3390/plants8100389 31575029 PMC6843125

[B69] Yiotis C. Manetas Y. (2010). Sinks for photosynthetic electron flow in green petioles and pedicels of zantedeschia aethiopica: evidence for innately high photorespiration and cyclic electron flow rates. Planta 232, 523–531. doi: 10.1007/s00425-010-1193-y 20490542

[B70] Zhang L. X. Paakkarinen V. van Wijk K. J. Aro E. M. (2000). Biogenesis of the chloroplast-encoded D1 protein: Regulation of translation elongation, insertion, and assembly into photosystem II. Plant Cell 12, 1769–1781. doi: 10.1105/tpc.12.9.1769 11006346 PMC149084

[B71] Zhao H. Zhou Q. Zhou M. Li C. Gong X. Liu C.. (2012). Magnesium deficiency results in damage of nitrogen and carbon cross-talk of maize and improvement by cerium addition. Biol. Trace Element Res. 148, 102–109. doi: 10.1007/s12011-012-9340-x 22294153

